# Supplementary material for: Content-rich biological network constructed by mining PubMed abstracts
Source: BMC Bioinformatics. 2004 Oct 8;5:147. doi: 10.1186/1471-2105-5-147 (PMC528731; doi:10.1186/1471-2105-5-147)
Supplement: Additional File 2 — The original results of the above study (non-essential files are deleted to keep the file size under the limit set by BMC bioinformatics). [file 1471-2105-5-147-S2.bz2 › chilibotAdditionalFile2/dip10/186ID8627325E631/html/ADA_ADORA1.html]

 


 **ADA** and **ADORA1** 
  
Found 67 abstracts in PubMed, retrieved 10.  
 

 What does Google say? 
 PDF only 
| .edu only 

---

**Interactive relationship** (e.g. stimulation, inhibition, etc)

**Inhibitory relationship**- The KCl induced release of 3H ACh, but not the release of 14C GABA, was potentiated when adenosine A1 receptor  [ **ADORA1** ]  activation was prevented by perfusing the cells with adenosine deaminase  [ **ADA** ]   **ADA**  or with dipropyl 8 cycloentylxanthine DPCPX.  Ref: 10661490 Brain Res, 2000
**Neutral relationship**

**Non-interactive relationship** (e.g. studied together, co-existance, homology, etc.)

- The post ATP facilitation could be prevented by the adenosine A1 receptor  [ **ADORA1** ]  antagonists 8 phenyltheophylline or dipropyl 8 cyclopentyltheophylline 50 nM, or superfusion of adenosine deaminase  [ **ADA** ] .  Ref: 11104829 Eur J Pharmacol, 2000
